# Improving the quantitative classification of Erlenmeyer flask deformities

**DOI:** 10.1007/s00256-020-03561-2

**Published:** 2020-07-30

**Authors:** Gautam Adusumilli, Joshua D. Kaggie, Simona D’Amore, Timothy M. Cox, Patrick Deegan, James W. MacKay, Scott McDonald, The GAUCHERITE Consortium

**Affiliations:** 1 Department of Radiology, University of Cambridge School of Clinical Medicine, Box 218, Cambridge Biomedical Campus, Cambridge, CB2 0QQ UK; 2 Present Address: St. Louis, USA; 3 Department of Medicine, Addenbrookes Hospital, University of Cambridge, Box 157, Hills Rd, Cambridge, CB2 0QQ UK

**Keywords:** MRI, Shape analysis, Erlenmeyer flask deformity, Surface area, Sphericity, Distal femur, Clinical cutoffs

## Abstract

The Erlenmeyer flask deformity is a common skeletal modeling deformity, but current classification systems are binary and may restrict its utility as a predictor of associated skeletal conditions. A quantifiable 3-point system of severity classification could improve its predictive potential in disease. Ratios were derived from volumes of regions of interests drawn in 50 Gaucher’s disease patients. ROIs were drawn from the distal physis to 2 cm proximal, 2 cm to 4 cm, and 4 cm to 6 cm. Width was also measured at each of these boundaries. Two readers rated these 100 femurs using a 3-point scale of severity classification. Weighted kappa indicated reliability and one-way analysis of variance characterized ratio differences across the severity scale. Accuracy analyses allowed determination of clinical cutoffs for each ratio. Pearson’s correlations assessed the associations of volume and width with a shape-based concavity metric of the femur. The volume ratio incorporating the metaphyseal region from 0 to 2 cm and the diametaphyseal region at 4–6 cm was most accurate at distinguishing femurs on the 3-point scale. Receiver operating characteristic curves for this ratio indicated areas of 0.95 to distinguish normal and mild femurs and 0.93 to distinguish mild and severe femurs. Volume was moderately associated with the degree of femur concavity. The proposed volume ratio method is an objective, proficient method at distinguishing severities of the Erlenmeyer flask deformity with the potential for automation. This may have application across diseases associated with the deformity and deficient osteoclast-mediated modeling of growing bone.

**Electronic supplementary material:**

The online version of this article (10.1007/s00256-020-03561-2) contains supplementary material, which is available to authorized users.

## Introduction

Erlenmeyer flask deformities (EFDs) are well-documented in a multitude of diseases with musculoskeletal involvement, including osteopetrosis, metaphyseal dysplasia, Gaucher’s, Niemann-Pick, and achondroplasia [1–3]. Impaired bone remodeling due to dysfunctional osteoclasts during skeletal growth leads to development an undertubulated distal femur, characterized by a loss of metaphyseal concavity and a wider metaphysis [4]. The deformity’s name comes from its resemblance to the lower portion of an Erlenmeyer flask, although the degree of undertubulation varies across patients.

Previous work has established a highly sensitive and specific means of objectifying the EFD through taking width measurements of the femur at certain distances from the physis and setting width ratios as a cutoff for the deformity [5]. A ratio > 0.58 between the width at 4 cm and the width at the physis was reported as being the most robust and accurate differentiator of EFD from non-EFD femurs against radiologist ratings. Notably, this differentiation was binary and did not account for the degree of undertubulation.

Under the current binary system, there have been weak associations of the presence or absence of EFD with other skeletal manifestations in disease such as osteonecrosis, osteosclerosis, and bone marrow infiltration [1]. Introducing a new, simple system that accounts for the degree of severity of EFD may uncover associations that enable a better understanding of skeletal diseases. Given the early stage of skeletal development that EFD manifests [1], this could have remarkable clinical utility for longitudinal patient monitoring and care plans.

Developing a method of quantifying and differentiating the deformity requires multiple criteria for maximal utility. It should, within the EFD positive patients, be able to differentiate mild and severe cases. It should be able to account for the loss of distal femur concavity in EFD, which has not been studied in the previous method involving width measurements. It should output analyzable, continuous clinical parameters that can be studied in the future against other skeletal manifestations in disease. And it should have the potential for semi-automation to derive the classification.

With these criteria as a premise, the aim of this study was to evaluate a new, more robust method of classifying EFD through volume ratios against radiologist ratings of “No EFD”, “Mild EFD”, “Severe EFD”. Additionally, width ratios were revisited as a means of classifying under this 3-point system and compared against our findings with volume ratios.

## Methods

### Image acquisition and demographics

The Gaucherite study was conducted under the approval of the East of England – Essex Research Ethics Committee (14/EE/1168) in the United Kingdom [6]. All procedures performed in studies involving human participants were in accordance with the ethical standards of the institutional and/or national research committee and with the 1964 Helsinki declaration and its later amendments or comparable ethical standards.

Informed consent was obtained from all individual participants included in the study. Consent to use data that was collected prior to the start of the Gaucherite study was obtained at patients’ normal NHS medical follow-ups. Patients signed to confirm that their imaging data could be used both retrospectively and prospectively as part of their normal clinical management. The consent form states the following: “I agree that my previously collected medical history, biological samples and images (such as MRI and DXA) and data collected from the analysis of this material can be used for the purposes of this research as described in this Information Sheet and stored for use for future research.”

Coronal T1-weighted 2D Fast Spin Echo (FSE) MRIs were acquired of diagnosed Gaucher’s patients using 1.5 T MRI systems between 2008 and 2017 for retrospective analysis. The voxel size was consistent across the study at 0.94 × 0.94 mm^2^, with a pixel bandwidth = ± 12/2 kHz, and with 1 average. Due to the nature of this study, imaging parameters varied over time and site, including repetition time (TR), which varied with values of 420, 460, 500, 520, 528, 540, 620, 640, and 800 ms. Similarly, the acquisition matrix consisted of multiple combinations of 256, 320, 384, or 512, with common schemes including 256 × 256, 256 × 384, 256 × 320, or 256 × 512. The receive coils included 8-channel knee coils, 8-channel lower body coils, and 32-channel cardiac coils. The slices had 1.5, 3, 4, 5, and 6 mm thicknesses. Other than slice thickness, the shape-based characteristics were chosen for analysis to reduce dependence on any MRI sequence.

MRI images of the distal femur and knee acquired under this protocol were evaluated in this study across 50 subjects, for a total of 100 femurs. There were 24 male and 26 female participants, of which 9 were Ashkenazi Jew, a population with a higher risk of Gaucher’s [7]. The age range was 23–78 years with a mean of 53 years.

### Volume and width ratio measurements of the distal femur

**Fig. 1:**
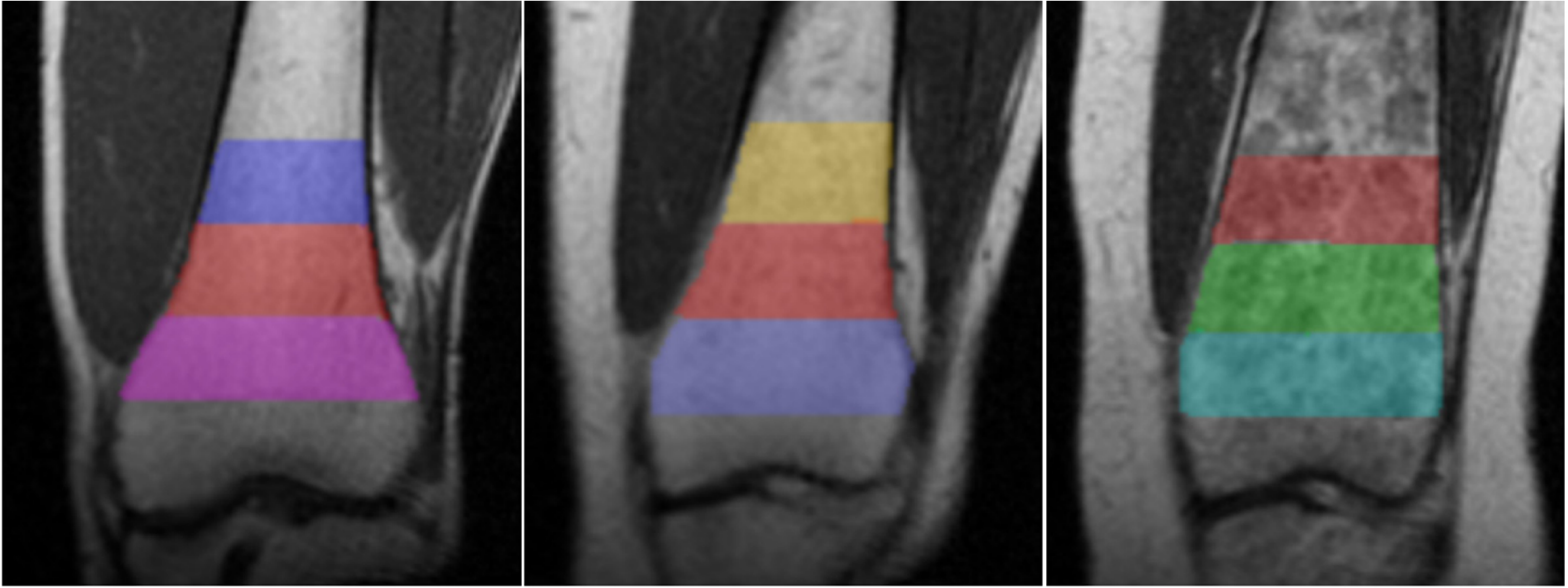
Distal femur ROIs to capture the Erlenmeyer flask deformity. Three ROIs were drawn for each femur: physis to 2 cm above the physis, 2 cm to 4 cm above the physis, and 4 cm to 6 cm above the physis. Note that each femur has a different degree of metaphyseal flaring. The left image is a femur rated “No EFD”, the center “Mild EFD”, and the right “Severe EFD”

The first phase of the study required drawing regions of interest (ROIs) on the distal femur with a standardized protocol. LIFEx (LIFEx Soft, v4.00 Orsay, France) was installed to collect ROIs from the DICOM image files, allow drawing and the measurement of distances within images (specific to that image), and output ROI volume measurements [8]. Selection of the slice for drawing included the following criteria: (1) physis clearly depicted, (2) > 15 cm distal femur + epiphysis length present, (3) no flexion deformities which indicate improper patient positioning [9], and (4) no image artifacts around the metaphysis. On the selected slice, the physis was identified and the first ROI was drawn utilizing the LIFEx drawing and distance measurement tools from the physis to 2 cm above. The second ROI was drawn from 2 cm above to 4 cm above, and the third ROI was drawn from 4 to 6 cm (Fig. 1). This process was repeated for both femurs across all patients. There were multiple instances of different slices being chosen for right vs. left femurs within the same patient due to patient positioning during the scan and subsequent image appearance.

Volume was automatically outputted as a variable by LIFEx upon completion of drawing. It must be noted that this metric of “Volume” is solely a reflection of the area of the femur within the ROI multiplied by slice thickness and does not reflect any further volume of the femur. The volume metrics for each ROI (0–2 cm from physis, 2–4 cm from physis, 4–6 cm from physis) were recorded. Absolute volume measurements were variable between patients due to varying slices thicknesses from 0.6 to 1.0 mm and the biological size of different femurs. However, taking the ratio of the volumes in our methodology accounted for these variations. Volume ratios were calculated for each possible volume combination: 2–4 cm/0–2 cm, 4–6 cm/0–2 cm, and 4–6 cm/2–4 cm.

Width measurements at the physis, 2 cm above, 4 cm above, and 6 cm above, were taken using the LIFEx distance measurement tool. Width ratios were calculated for each possible distance combination, at 2 cm/physis, 4 cm/physis, 6 cm/physis, 4 cm/2 cm, 6 cm/2 cm, and 6 cm/4 cm.

### Radiologist classification of the Erlenmeyer flask deformity

The second phase of the study was radiologist classification of the femurs for the EFD. Two readers, one with 12 years of experience in musculoskeletal radiology (R1) and one with 4 years of experience (R2), independently reviewed each femur across multiple slices according to standard radiological practice and rated the femurs by “No EFD”, “Mild EFD”, and “Severe EFD”. Intra-rater reliability was assessed on R2 by requesting a repeat of the rating process after 2 weeks.

### Statistical analysis

PSPP was used as the software for statistical analysis. Inter-rater reliability between R1 and R2 in the 3-point rating system of EFD was determined using linear-weighted kappa with 95% confidence intervals (CI). Intra-rater reliability of R2 was also assessed by linear-weighted kappa with 95% CI.

Further analyses were conducted only on femurs with concordant ratings by R1 and R2. The data was checked for normality and the one-way analysis of variance (ANOVA) was conducted between the “No EFD”, “Mild EFD”, and “Severe EFD” groups to detect significant differences in width ratios and volume ratios across groups. Box-and-whisker plots were created for the three volume ratio parameters to visually depict differences in median and IQR between the three EFD rating groups. As a sub-analysis, when two readers concordantly rated the two femurs within the same patient differently, the average and standard deviation of volume ratio difference between femurs were calculated.

Based on the initial findings, cutoffs were determined for each of the volume ratio parameters between the three degrees of EFD. Accuracy analyses and receiver operating characteristic (ROC) curves were used to compare proficiency of each ratio at their respective cutoffs. This was repeated for each of the ANOVA-significant width ratio parameters. ROC curves for these width ratios were compared against the ROC curves for the volume ratios.

Finally, the ability of width ratios and volume ratios to account for the concavity of the femur was compared. This is the very basis of radiologist classification, as visually more severe EFD femurs have a greater loss of concavity (Fig. 1). The ROIs drawn for each femur were combined into a single ROI (0–6 cm from physis) and a metric called “SHAPE_Sphericity” was outputted from these ROIs using the LIFEx 4.00 built-in code for shape analysis. “SHAPE_Sphericity” is defined by LIFEx 4.00 as how spherical an ROI is, with a value of 1 indicating completely spherical [8]. Using Pearson’s correlation analysis, this metric was associated with the absolute width measurements at the physis, 2 cm, 4 cm, and 6 cm and volume measurements at 0–2 cm, 2–4 cm, and 4–6 cm, to evaluate for significant associations and characterize the strength of the associations.

## Results

A total of 300 ROIs were drawn for the 100 femurs in this study. Figure 1 depicts examples of ROIs at different degrees of the EFD.

### Radiologist classification—inter-rater reliability

Table 1 depicts the spread of EFD ratings across the 3-point system and allows a subjective assessment of concordance between R1 and R2.

**Table 1:** Comparison of radiologist ratings of the Erlenmeyer flask deformity across a 3-point system of classification

		Radiologist 2			
		No EFD	Mild EFD	Severe EFD	Total
Radiologist 1	No EFD	19	1	0	20
	Mild EFD	8	31	3	42
	Severe EFD	0	4	34	38
	Total	27	36	37	100

The linear-weighted kappa statistic between R1 and R2 was 0.81 with a 95% CI of 0.72 to 0.90, indicating a substantial to almost perfect agreement between raters in the classification of EFD [10].

Notably, there were 10 patients in which both raters gave two distinct EFD classifications within the same patient bilaterally (i.e. right femur mild, left femur severe) and the raters matched exactly in 9 of those patients.

### Radiologist classification—intra-rater reliability

The linear-weighted kappa statistic between R2’s ratings at time point 1 and 2 weeks later at time point 2 was 0.86 with a 95% CI of 0.79 to 0.94. This indicated a substantial to almost perfect intra-rater reliability in the classification of EFD [10].

### Volume and width ratios across EFD severities

Only the 84 femurs with concordant radiologist ratings of EFD severity were evaluated. One-way ANOVA was conducted to determine the degree to which volume ratios and width ratios could differentiate among EFD severity (Table 2). Post hoc analysis with Tukey’s HSD was conducted for the significant results.

**Table 2:** One-way ANOVA of Erlenmeyer flask deformity severity against volume and width ratios. Ratios with non-overlapping confidence intervals are in italics. Tukey’s HSD is reported with “EFD 0/1” representing the comparison between groups with EFD Severity Rating 0 and EFD Severity Rating 1, and similarly for the “EFD 0/2” and “EFD 1/2”

Ratios	EFD Severity Rating (0 = normal, 1 = mild, 2 = severe)	95% confidence interval	F-statistic	*p* value	Tukey’s HSD
$\frac{Volume\ 4-6\ cm}{Volume\ 0-2\ cm}$	0	0.50–0.55	119.02	< 0.0001	EFD 0/1: 0.099 (*p* < 0.0001)
	1	0.61–0.64			EFD 0/2: 0.202 (*p* < 0.0001)
	2	0.71–0.75			EFD 1/2: 0.103 (*p* < 0.0001)
$\frac{\ \mathrm{Volume}\ 4-6\ \mathrm{cm}}{\mathrm{Volume}\ 2-4\ \mathrm{cm}}$	0	0.74–0.79	23.04	< 0.001	EFD 0/1: 0.065 (*p* < 0.001)
	1	0.80–0.85			EFD 0/2: 0.103 (*p* < 0.0001)
	2	0.85–0.88			EFD 1/2: 0.038 (*p* < 0.05)
$\frac{Volume\ 2-4\ cm}{Volume\ 0-2\ cm}$	0	0.68–0.71	78.61	< 0.0001	EFD 0/1: 0.067 (*p* < 0.0001)
	1	0.75–0.77			EFD 0/2: 0.153 (*p* < 0.0001)
	2	0.83–0.86			EFD 1/2: 0.086 (*p* < 0.0001)
$\frac{\mathrm{Width}\ 2\ \mathrm{cm}}{\mathrm{Width}\ \mathrm{Physis}}$	0	0.70–0.77	20.41	< 0.001	EFD 0/1: not significant
	1	0.74–0.78			EFD 0/2: 0.115 (*p* < 0.0001)
	2	0.82–0.88			EFD 1/2: 0.093 (*p* < 0.0001)
$\frac{\mathrm{Width}\ 4\ \mathrm{cm}}{\mathrm{Width}\ \mathrm{Physis}}$	0	0.49–0.57	51.89	< 0.001	EFD 0/1: 0.055 (*p* < 0.05)
	1	0.57–0.60			EFD 0/2: 0.194 (*p* < 0.0001)
	2	0.69–0.75			EFD 1/2: 0.139 (*p* < 0.0001)
$\frac{Width\ 6\ cm}{ Width\ Physis}$	0	0.39–0.47	54.25	< 0.001	EFD 0/1: 0.082 (*p* < 0.001)
	1	0.49–0.53			EFD 0/2: 0.211 (*p* < 0.0001)
	2	0.61–0.67			EFD 1/2: 0.129 (*p* < 0.0001)
$\frac{\mathrm{Width}\ 4\ \mathrm{cm}}{\mathrm{Width}\ 2\ \mathrm{cm}}$	0	0.69–0.75	37.79	< 0.001	EFD 0/1: 0.054 (*p* < 0.01)
	1	0.75–0.80			EFD 0/2: 0.13 (*p* < 0.0001)
	2	0.83–0.87			EFD 1/2: 0.077 (*p* < 0.0001)
$\frac{Width\ 6\ cm}{Width\ 2\ cm}$	0	0.55–0.61	32.85	< 0.001	EFD 0/1: 0.096 (*p* < 0.001)
	1	0.65–0.71			EFD 0/2: 0.170 (*p* < 0.0001)
	2	0.73–0.77			EFD 1/2: 0.074 (*p* < 0.001)
$\frac{\mathrm{Width}\ 6\ \mathrm{cm}}{\mathrm{Width}\ 4\ \mathrm{cm}}$	0	0.78–0.84	11.68	< 0.001	EFD 0/1: 0.066 (*p* < 0.001)
	1	0.85–0.90			EFD 0/2: 0.076 (*p* < 0.0001)
	2	0.87–0.90			EFD 1/2: not significant

**Fig. 2:**
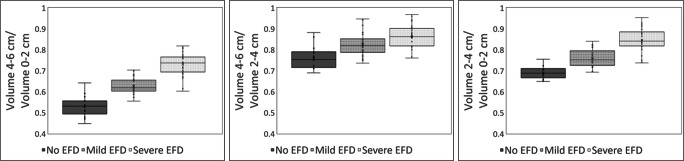
Box-and-whisker plots of the three volume ratios across the three EFD severities. **a** The plot for the ratio “volume 4–6 cm/volume 0–2 cm”. **b** “volume 4–6 cm/volume 2–4 cm”. **c** “volume 2–4 cm/volume 0–2 cm”

Box-and-whisker plots were created to visually depict the spread of each volume ratio across the three degrees of EFD severity (Fig. 2).

### Distinct femur ratings within the same patient

The nine patients with rater-concordant, distinct EFD severites between the right and left femurs were evaluated. All severity differences were only of one degree (i.e., there were no patients with one severe and one normal EFD femur). The average difference in ratios between no EFD vs. mild EFD and mild EFD vs. severe EFD is presented in Table 3.

**Table 3:** Volume ratios and their ability to distinguish varying severity of the Erlenmeyer flask deformity across femurs within the same patient

	Number of patients	Average difference in (volume 4–6 cm/volume 0–2 cm) ± SD	Average difference in (volume 4–6 cm/volume 2–4 cm) ± SD	Average difference in (volume 2–4 cm/volume 0–2 cm) ± SD
No EFD + mild EFD	3	0.12 ± 0.05	0.07 ± 0.05	0.08 ± 0.07
Mild EFD + severe EFD	6	0.08 ± 0.04	0.03 ± 0.05	0.07 ± 0.03
Total	9	0.09 ± 0.04	0.05 ± 0.05	0.07 ± 0.04

*SD* standard deviation

Note that from a less severe to more severe femur within the same patient, the ratio increased in all cases.

### Width and volume clinical cutoffs for EFD

Cutoffs were first determined for width ratios. ROC curves were analyzed for each ratio between normal to mild severity to set cutoffs maximizing sensitivity (thus minimizing false negatives) with minimal impact on specificity. ROC curves to set cutoffs between mild and severe EFD were then analyzed to maximize specificity and reduce false positives, with minimal impact on sensitivity. This approach targeted the accurate distinction of mild EFD cases from normal femurs or severe EFD.

Overall accuracy of each cutoff was assessed using only femurs rated on either side of the cutoff (i.e., severe EFD cutoff was evaluated using only mild and severe EFD femurs). Note that only width ratios with accuracies > 70% for both normal to mild and mild to severe distinctions are presented here in Table 4. The remaining width ratios were not as proficient at distinguishing severity of EFD and can be found in Supplementary Table 1. Notably, 0.58 was discussed as the cutoff for no EFD vs. EFD in the previous study under the “Width 4 cm/Width Physis” ratio [5]. The cutoff for the same width ratio in this study was 0.55 for no EFD vs. mild EFD cases (Supplementary Table 1).

**Table 4:** Accuracy analyses of width-ratio-based clinical cutoffs at differentiating severities of the Erlenmeyer flask deformity, with the best performing ratio in italics

Ratio	Severity cutoff	Accuracy (95% CI)
$\frac{\ Width\ 6\ cm}{Width\ Physis}$	Normal ≥ mild (cutoff = 0.47)	Sensitivity: 80.7% (62.5–92.6%)
		Specificity: 79.0% (54.4–94.0%)
		PPV: 86.2% (72.0–93.8%)
		NPV: 71.4% (54.0–84.2%)
		Accuracy: 80.0% (66.3–90.0%)
	Mild ≥ Severe (cutoff = 0.61)	Sensitivity: 73.5% (55.6–87.1%)
		Specificity: 93.6% (78.6–99.2%)
		PPV: 92.6% (76.3–98.0%)
		NPV: 76.3% (64.6–85.0%)
		Accuracy: 83.1% (71.7–91.2%)
$\frac{\ \mathrm{Width}\ 4\ \mathrm{cm}}{\mathrm{Width}\ 2\mathrm{cm}}$	Normal ≥ mild (cutoff = 0.75)	Sensitivity: 77.4% (58.9–90.4%)
		Specificity: 79.0% (54.4–94.0%)
		PPV: 85.7% (71.1–93.6%)
		NPV: 68.2% (51.8–81.1%)
		Accuracy: 78.0% (64.0–88.5%)
	Mild ≥ severe (cutoff = 0.82)	Sensitivity: 76.5% (58.8–89.3%)
		Specificity: 83.9% (66.3–94.6%)
		PPV: 83.9% (69.5–92.2%)
		NPV: 76.5% (63.5–85.9%)
		Accuracy: 80.0% (68.2–88.9%)
$\frac{\ \mathrm{Width}\ 6\ \mathrm{cm}}{\mathrm{Width}\ 2\mathrm{cm}}$	Normal ≥ mild (cutoff = 0.61)	Sensitivity: 83.9% (66.3–94.6%)
		Specificity: 73.7% (48.8–90.9%)
		PPV: 83.9% (70.7–91.8%)
		NPV: 73.7% (54.6–86.7%)
		Accuracy: 80.0% (66.3–90.0%)
	Mild ≥ severe (cutoff = 0.72)	Sensitivity: 67.7% (49.5–82.6%)
		Specificity: 74.2% (55.4–88.1%)
		PPV: 74.2% (60.2–84.5%)
		NPV: 67.7% (55.2–78.0%)
		Accuracy: 70.8% (58.2–81.4%)

*PPV* positive predictive value, *NPV* negative predictive value

Translatability of volume ratios to clinical cutoffs was assessed next. Cutoffs optimizing sensitivity between the Normal and Mild distinction and specificity between the Mild and Severe distinction were determined by ROC curves. The accuracy analyses are presented in Table 5.

**Table 5:** Accuracy analyses of volume-ratio-based clinical cutoffs at differentiating severities of the Erlenmeyer flask deformity, with the best performing ratio in italics

Ratio	Severity cutoff	Accuracy (95% CI)
$\frac{Volume\ 4-6\ cm}{Volume\ 0-2\ cm}$	Normal ≥ mild (cutoff = 0.57)	Sensitivity: 96.8% (83.3–99.9%)
		Specificity: 84.2% (60.4–96.6%)
		PPV: 90.9% (77.9–96.6%)
		NPV: 94.1% (69.7–99.1%)
		Accuracy: 92.0% (80.8–97.8%)
	Mild ≥ severe (cutoff = 0.68)	Sensitivity: 82.4% (65.5–93.2%)
		Specificity: 90.3% (74.3–98.0%)
		PPV: 90.3% (75.9–96.5%)
		NPV: 82.4% (69.1–90.7%)
		Accuracy: 86.2% (75.3–93.5%)
$\frac{\ \mathrm{Volume}\ 4-6\ \mathrm{cm}}{\mathrm{Volume}\ 2-4\ \mathrm{cm}}$	Normal ≥ mild (cutoff = 0.79)	Sensitivity: 77.4% (58.9–90.4%)
		Specificity: 73.7% (48.8–90.9%)
		PPV: 82.8% (68.8–91.3%)
		NPV: 66.7% (49.7–80.2%)
		Accuracy: 76.0% (61.8–86.9%)
	Mild ≥ severe (cutoff = 0.85)	Sensitivity: 61.8% (43.6–77.8%)
		Specificity: 71.0% (52.0–85.8%)
		PPV: 70.0% (55.9–81.1%)
		NPV: 62.9% (51.1–73.3%)
		Accuracy: 66.2% (53.4–77.4%)
$\frac{\ \mathrm{Volume}\ 2-4\ \mathrm{cm}}{\mathrm{Volume}\ 0-2\ \mathrm{cm}}$	Normal ≥ mild (cutoff = 0.72)	Sensitivity: 83.9% (66.3–94.6%)
		Specificity: 79.0% (54.4–94.0%)
		PPV: 86.7% (72.9–94.0%)
		NPV: 75.0% (56.5–87.4%)
		Accuracy: 82.0% (68.6–91.4%)
	Mild ≥ severe (cutoff = 0.80)	Sensitivity: 76.5% (58.8–89.3%)
		Specificity: 96.8% (83.3–99.9%)
		PPV: 96.3% (78.9–99.5%)
		NPV: 79.0% (67.1–87.3%)
		Accuracy: 86.2% (75.3–93.5%)

Subsequently, two ROC curves were obtained for each of the ratios for the classification of the two degrees of distinction. Among all ratios, the volume ratios of 4–6 cm/0–2 cm and 2–4 cm/0–2 cm had the largest area under the curve (AUC) for the no EFD vs. mild EFD distinction (0.95 and 0.92, respectively) as well as the mild EFD vs. severe EFD distinction (0.93 and 0.90, respectively).

The width ratios of 4 cm/physis and 6 cm/physis showed strong AUCs of 0.92 and 0.89 for the mild vs. severe distinction but performed worse at classifying between no EFD and mild EFD with AUCs of 0.74 and 0.80, respectively.

### Concavity of the femur predicted by volume and width ratios

To better understand the distinctness of information that volume and width capture at classifying degrees of EFD severity, a Pearson’s correlation analysis on the association of our width and volume measurements with femur sphericity was conducted as described in the “Methods”.

All three volume measurements had significant (*p* < 0.01) and moderate Pearson’s correlations with sphericity of − 0.42, − 0.39, and − 0.37, respectively. The width measurements showed negligible correlations ranging from − 0.03 to 0.04. Notably, the volume associations were directionally valid, as a greater metaphyseal volume seen in EFD indicates loss of concavity and thereby sphericity.

## Discussion

The aim of this study was to introduce a 3-point severity scale of EFD classification and evaluate the proficiency of volume ratios at classifying under this scale, compared against width ratios. Our findings demonstrate that volume ratios incorporating both the metaphysis and a diametaphyseal region can best delineate severities of EFD and tend to be more proficient than width ratios because they account for loss of distal femur concavity. Several clinical cutoffs based on these volume ratios had high ROC AUCs, suggesting their potential utility among radiologists to accurately classify EFD severity. Thus, the degree of EFD severity may be characterized early in disease and studied more robustly as a predictor of further skeletal manifestations.

Our 3-point system of EFD classification is functionally simple and the concordance between our two raters trained at different institutions with different levels of experience indicates that the system does indeed capture meaningful, discernible differences. Among the ratios, “volume 4–6 cm/volume 0–2 cm” lends clinical cutoffs that can best classify EFD objectively on this scale, ensure the accurate detection of mild cases of EFD, and may even be used to distinguish variable femurs within the same patient. The remaining ratios would be inefficient to use, as they are less proficient at differentiating at least one of the two thresholds. “Volume 2–4 cm/volume 0–2 cm”, for example, is weaker at differentiating normal from mild femurs, suggesting that the distinction between these severities may be diametaphyseal flaring further from the physis. Similarly, the relative inability of “volume 4–6 cm/volume 2–4 cm” to classify EFD accurately may be due to its lack of metaphyseal volume information. The overall weaker performance of width ratios, especially with the normal to mild distinction, is also notable and may be explained by their inability to detect loss of concavity.

While the presence of EFD has been weakly associated with other skeletal markers in diseases such as Gaucher’s [1], multiple studies have discussed that osteoclast impairment, the very cause of EFD, is also indicated in other forms of bone loss and immune system crosstalk [4, 11, 12]. Clinicians under the current binary system of EFD classification may be missing information that could improve the predictive value of EFD, and this information could be introduced through the 3-point severity scale we have presented in this study. Width ratios were previously shown across many cases to be highly accurate at EFD classification under a binary system [5], but have underperformed under our 3-point system. From our findings across a comparably large sample of femurs, we conclude that “volume 4–6 cm/volume 0–2 cm” is the metric with the most utility for EFD severity distinction and argue that subtle concavity changes between normal and mild EFD are most likely to be missed under previous methods of classification.

In the context of skeletal radiology practice and research, application of our method and derivation of the proposed volume metric would enable objective and robust EFD classification, provide a continuous clinical parameter for analyses, and allow researchers to better understand diseases causing EFD. Although this study was conducted on a Gaucher’s patient base, it may be extrapolated to other diseases with EFD resulting from bone marrow expansion, as the deformity has been reported to be consistent across these diseases (e.g., membranous lipodystrophy, Thalassemia, Niemann-Pick) [1]. The findings of this study would thereby have utility across a large number of cases.

Several study limitations are worth noting. Clinical cutoffs by ROC curves were set to optimize the accurate detection of mild EFD femurs, which have been least reported on and may therefore be the most interesting to follow longitudinally. However, further work may prove otherwise, and these clinical cutoffs may therefore be subject to change. Additionally, 16 patients with discordant radiologist ratings of EFD severity were excluded from the analysis due to an inability to stratify into groups, and this may have led to an overestimation of the ability of the AUCs to distinguish between severities. Thirdly, MRI images are subject to errors based on patient positioning [13] and may result in inaccurate determinations of volume and width. Because EFD remains static after a certain age, we were able to reduce error by choosing patient scan dates with the highest-quality images. Nonetheless, this may be a source of some measurement errors. Finally, a study design limitation was incurred by the subjective selection of the slice used to measure width and volume. Patients often had more than one slice that suited our protocol criteria, but we sought to reduce error by selecting slices with femur cuts that appeared consistent across patients.

The findings of this study may be enhanced by automating the segmentation of volumes and derivation of ratios. Automatic segmentation of the femur has been widely reported on in recent years and indicates strong potential for our volume ratio measurements to become automated [14–18]. This meets the last of the criteria we set forth for this method. Future studies should therefore seek the development of a methodology to automatically segment distal femur ROIs for the extraction of volume. Clinicians may eventually be able to input an image and instantly receive information about the degree of EFD severity, both as a continuous variable through volume ratio and as an ordinal rating.

In conclusion, we report upon a new measurement method based on volume ratios at the distal femur to distinguish the Erlenmeyer flask deformity on a 3-point scale of severity. The proposed method is highly proficient, accounts for the concavity of the distal femur, produces an assessable continuous clinical variable, and has strong potential for automation. Radiologists studying and rating this deformity in bone marrow expansion diseases would benefit the most in the future from our findings.

## Electronic supplementary material

ESM 1 (DOCX 25 kb)
