# Supplementary material for: Improving the quantitative classification of Erlenmeyer flask deformities
Source: Skeletal Radiol. 2020 Jul 30;50(2):361–9. doi: 10.1007/s00256-020-03561-2 (PMC7736022; doi:10.1007/s00256-020-03561-2)
Supplement: Supplementary file 1 — (DOCX 25 kb) [file 256_2020_3561_MOESM1_ESM.docx]

## Supplementary Tables and Figures

**Supplementary Table 1.**

| $\frac{Width 2 cm}{Width Physis}$ | Normal -> Mild (cutoff = 0.75) | Sensitivity: 64.5% (45.4 – 80.8%)  Specificity: 57.9% (33.5 – 79.8%)  PPV: 71.4% (58.1 – 81.8%)  NPV: 50.0% (35.2 – 64.8%)  Accuracy: 62.0% (47.2 – 75.4%) |
| --- | --- | --- |
|  | Mild -> Severe (cutoff = 0.80) | Sensitivity: 67.7% (49.5 – 82.6%)  Specificity: 90.3% (74.3 – 98.0%)  PPV: 88.5% (71.8 – 95.8%)  NPV: 71.8% (60.7 – 80.8%)  Accuracy: 78.5% (66.5 – 87.7%) |
| $\frac{Width 4 cm}{Width Physis}$ | Normal -> Mild (cutoff = 0.55) | Sensitivity: 74.2% (55.4 – 88.1%)  Specificity: 63.2% (38.4 – 83.7%)  PPV: 76.7% (63.8 – 86.0%)  NPV: 60.0% (43.0 – 74.9%)  Accuracy: 70.0% (55.4 – 82.1%) |
|  | Mild -> Severe (cutoff = 0.69) | Sensitivity: 70.6% (52.5 – 84.9%)  Specificity: 96.8% (83.3 – 99.9%)  PPV: 96.0% (77.5 – 99.4%)  NPV: 75.0% (64.0 – 83.5%)  Accuracy: 83.1% (71.7 – 91.2%) |
| $\frac{Width 6 cm}{Width 4 cm}$ | Normal -> Mild (cutoff = 0.83) | Sensitivity: 71.0% (52.0 – 85.8%)  Specificity: 63.2% (38.4 – 83.7%)  PPV: 75.9% (62.6 – 85.5%)  NPV: 57.1% (41.1 – 71.8%)  Accuracy: 68.0% (53.3 – 80.5%) |
|  | Mild -> Severe (cutoff = 0.88) | Sensitivity: 61.8% (43.6 – 77.8%)  Specificity: 61.3% (42.2 – 78.2%)  PPV: 63.6% (51.0 – 74.6%)  NPV: 59.4% (46.7 – 70.9%)  Accuracy: 61.5% (48.6 – 73.4%) |
